# A year in review – evaluating the launch of IJTLD OPEN

**DOI:** 10.5588/ijtldopen.24.0613

**Published:** 2025-01-01

**Authors:** H.D. Blackbourn, G.B. Migliori

**Affiliations:** ^1^ International Union Against Tuberculosis and Lung Disease, Paris, France;; ^2^ Istituti Clinici Scientifici Maugeri, Istituto di Ricovero e Cura a Carattere Scientifico, Tradate, Italy.

**Keywords:** open access, *International Journal of Tuberculosis and Lung Disease*, The Union, cOAlition S

## Abstract

One year on from the launch of our new open access (OA) journal, IJTLD OPEN, we review its impact. Similar to our flagship journal, the IJTLD, articles published in IJTLD OPEN span a range of topics related to lung health and the majority focus on TB in low- to middle-income countries (LMICs). Interestingly, there has been no lag period in readers accessing the content, with downloads for IJTLD OPEN soon matching and exceeding those for the IJTLD. This demonstrates that OA is helping us to achieve our goal of improving knowledge dissemination in LMICs, where there is restricted access to subscription journals. Citation analysis of the first few issues of IJTLD OPEN also suggests that this higher level of downloads is leading to articles being cited at an accelerated rate.

The launch of a new journal is a time of excitement, tinged with nervousness. You hope that your analysis is accurate – and that you have judged the timing correctly – but there is always a level of uncertainty. Will the community embrace your vision for this new journal? This is the position we found ourselves in January 2024, with the launch of IJTLD OPEN, a Plan S compliant open access (OA) journal.^1,2^ One year on, we are delighted to report that the launch has exceeded our expectations.

The priority for any new journal is ensuring the integrity of its content. As such, we are grateful to our Editorial Board for kindly agreeing to review content for both the IJTLD and IJTLD OPEN.^3^ This avoided the risk that the quality of our OA articles might be compromised, as has been the case for some commercial publishers of OA journals.^4^ The next priority was to attract good authors. Fortunately, the IJTLD has an established reputation, and its impact factor has been rising in recent years, so we were able to appeal to existing authors and those mandated to publish in an OA journal. As a consequence, we were able to publish articles from experts on a wide range of topics, but with TB in low to middle income countries (LMICs) as the mainstay.^5–9^

A key objective of the Union is knowledge dissemination. The traditional subscription-based publishing model is challenging for an organisation such as ours, which focuses on lung health in LMICs. Such settings have limited funds for journal subscriptions, so access is severely restricted. Having published several OA articles in recent years, including the IJTLD Clinical Standards for Lung Health,^10,11^ it appeared that OA is effective at driving knowledge dissemination, and so it has been proven for IJTLD OPEN. However, we had anticipated a significant lag period before article downloads approached the levels seen for the IJTLD while the community became aware of this new journal. Instead, downloads for IJTLD OPEN soon matched the full-text downloads for the equivalent issue of the IJTLD (see Figure). In July 2024, there was a further marked increase in downloads as the articles appeared on PubMed Central (PMC). This trend for increased downloads has continued throughout 2024 and shows no sign of abating. Furthermore, because authors retain copyright, as part of our publishing process, we send each corresponding author a copy of their published PDF to use as they see fit (e.g., to send to colleagues, post on ResearchGate or display on institutional websites). Although such usage is not measured directly, it will also help to ensure the free dissemination of knowledge.

**Figure.**
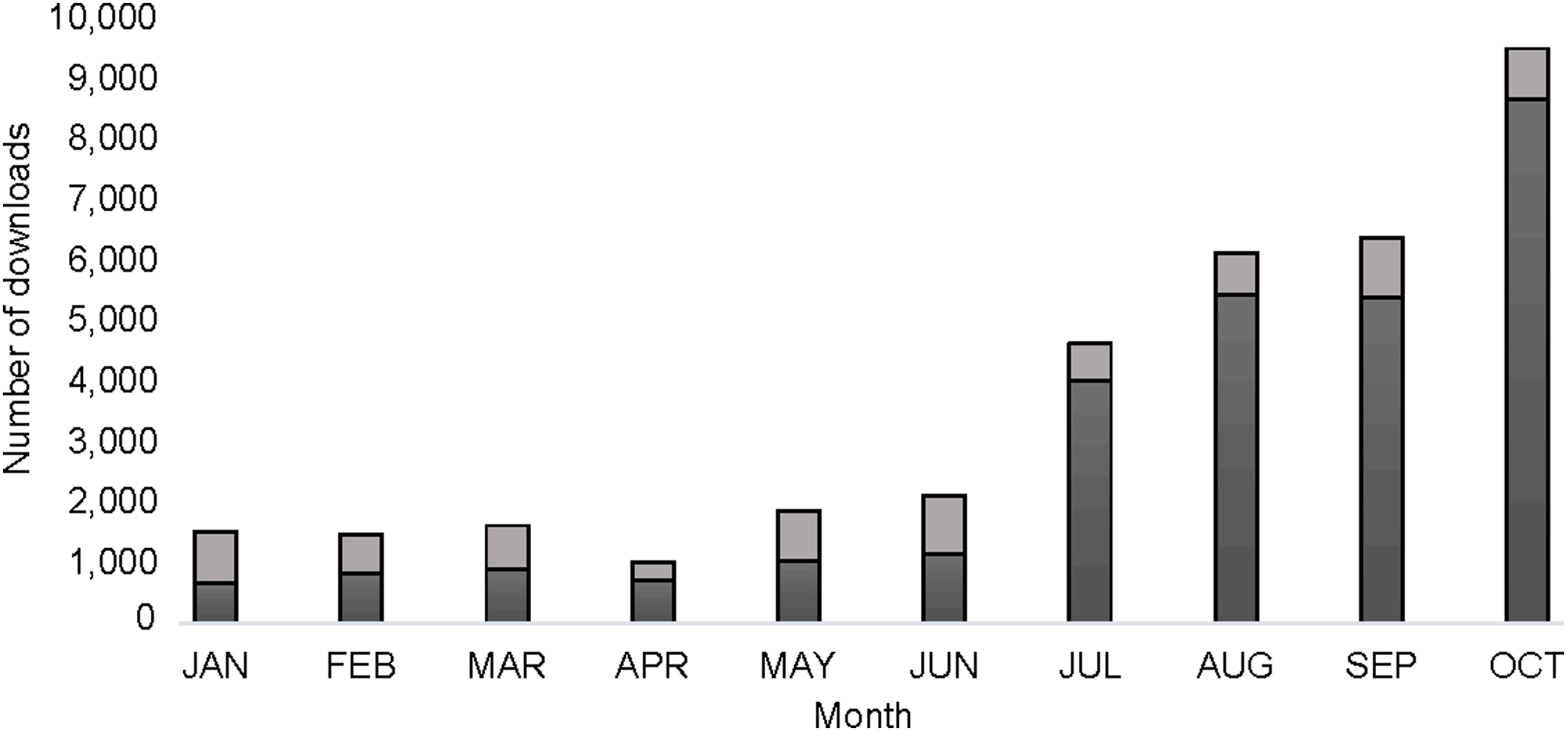
Comparison of full-text downloads from January to October 2024 for issues of IJTLD OPEN (black bars) vs the IJTLD (grey bars). The marked increase in downloads of IJTLD OPEN in July corresponds to the journal being made available on PubMed Central. Please note that data for the IJTLD does not include the extensive monthly downloads for the archive.

It has long been recognised that OA drives an increase in downloads, but the relationship to citations is less clear.^12^ We are in the interesting position of now being able to directly compare a subscription journal with an open access journal – each journal having the same scope with articles reviewed by the same Editorial Board. Although there is typically a delay of 6–12 months before articles are cited to any meaningful extent in the IJTLD, we have noted a difference for IJTLD OPEN. Early data indicates that articles published in IJTLD OPEN are being cited at an accelerated rate compared to the IJTLD. This is in keeping with a recent study, which reported that OA leads to citations from a broader range of research users.^13^ We stress that this is only a preliminary finding, but given the heightened visibility of IJTLD OPEN articles on publishing and institutional websites, it is logical to assume that the pattern will continue. We will report more on this once further data is available.

In summary, it is gratifying to know that our efforts – and those of our authors, our Editorial Board and reviewers – are having such a demonstrable impact. We conclude that OA is the optimal model for knowledge dissemination to a global audience, and in particular for those based in LMICs. The Union is committed to continuing to subsidise the cost of publishing for our authors, but we call on funding agencies to offer further OA support for authors in these settings, so that their insights can be shared.
